# Early versus conventional closure of intestinal stoma: Insights from a tertiary care hospital in India

**DOI:** 10.6026/973206300211493

**Published:** 2025-06-30

**Authors:** Manish Kumar, Ashutosh Patel, Amal Pushp Singh, Sunil Kumar, Narain C.M.

**Affiliations:** 1 Department of General Surgery, Netaji Subhas Medical College and Hospital, Amhara Bihta, Patna, Bihar, India; 2 Department of General Surgery, AIIMS, Patna, Bihar, India

**Keywords:** Early intestinal stoma closure, conventional intestinal stoma closure

## Abstract

Outcomes of stoma closure surgery (a surgical procedure to reconnect the bowel and close a stoma) in 110 patients at the Department of General Surgery at Netaji Subhas Medical College and Hospital, Patna is of interest. 55 patients in the ECS (early stoma closure) group received treatment within 14-28 days, whereas 55 patients in the CSC (conventional stoma closure) group had treatment after 8-12 weeks. There were fewer complications in the ECS group (44%) than in the CSC group (64%). Thus, problems with stoma closure surgery were less often in ECS (4%) than in CSC (6%). Results suggest that early stoma closure can be performed safely in patients who fit the requirements.

## Background:

In developing nations, ileostomies are still frequently performed in emergency surgical environments where individuals report to doctors late in their medical conditions, preventing primary closure and conditions that are infectious such as gastrointestinal or tubercular punctures are prevalent [1, 2- 3]. In developed nations, ileostomies are primarily constructed as a barrier of protection for anastomosis distal colorectal or ileoanal pouch. Patients react variably to the development of stomas and these reactions include changes in way of life, sexual orientation and everyday activities as well as impressions of an altered image of themselves [2, 3, 4- 5]. Stoma formation, on the contrary hand, is a therapy that cures illness, reduces pain and enhances health; it could additionally have a beneficial effect [6-7]. A person's ability to adjust to daily life with a stoma depends on a number of personal characteristics, which includes age, socioeconomic status, attitude and gender. Research has indicated that people who have a stoma had a lower quality of life than those who had similar procedures done devoid of stoma development [8, 9- 10]. While understanding that the circumstance was transitory may hinder adjustment to dealing with a transient ileostomy [8], reversing the temporary stoma improved quality of life [11, 12-13]. The main purpose of a defuncting stoma is to shield the anastomosis and avoid pelvic infection following intestinal resection. Provisional ileostomy is linked to less anastomotic leaks, according to a Cochrane analysis [14, 15-16]. A non-functional stoma lessens the requirement for an immediate reoperation [15, 16-17]. In general, stoma repair is done eight to twelve weeks later. However, throughout this time, stoma-related issues impact standard of life quality (QoL) [18, 19-20]. Early provisional stoma closure may lessen discomfort for patients and stoma-related complications. Early stoma closure after bowel surgery is a safe and effective approach that enhances quality of life without increasing complications or hospital stay [21]. Early stoma closure does not increase the risk of surgery or hospitalisation, but it significantly improves the quality of life [22]. It is possible to reverse a temporary stoma 8-10 days postoperatively, although there will be more wound problems [23]. According to other research, there was no discernible difference in death and morbidity between the stages of stoma repair [13, 14-15]. Intestinal continuity repair is typically linked to a reduced death rate. The reports on early versus conventional stoma closure are conflicting [19, 20-21]. However, stoma inversion may result in a second operation due to significant complications that range from zero percent to nine percent and minor side effects that range from four percent to thirty percent [16, 17-18]. Therefore, it is of interest to compare early and conventional stoma closure following bowel surgery.

## Methods and Materials:

110 consecutive patients between the ages of 18 years and 70 years who underwent temporary stoma following bowel surgery both in elective and emergency setting, irrespective of the indication for primary surgery were included in the study. Patients in whom emergency stoma revision was done for necrosis or gangrene, those with evidence of sepsis or organ failure in the postoperative course, any radiological signs of primary anastomotic leak evident on water soluble contrast examination before stoma closure and patients with poor nutritional status (Hb<8 g% and Albumin < 2.5 g%) were excluded from the study. Patients with one or more comorbidities like diabetes, hypertension and others were stabilized well before the stoma closure surgery.

## Early stoma closure (ECS):

This group included 55patients who had stoma closure performed 14-28 days after index surgery. ESC was not performed during the same hospital stay since the vast majority of patient's required immediate surgery for the index resection. As a result, most patients were readmitted following stoma closure stability.

## Conventional stoma closure (CSC):

This group included 55patients whose temporary stomas were closed between 8 and 12 weeks in accordance with our hospital's unit procedure. Following general/spinal surgical anesthesia, the short-term stoma was closed by incision of the peristomal skin, mobilization and a sutured connection by hand-sewn interrupted approach in two layers. As is standard procedure, the inner layer was seromuscularly lined with polyglactin 2 0 and the outer layer was sero-muscularly lined with Silk 2 0. Given the expense, stapled anastomosis was performed in very few instances. In accordance with unit procedure, postoperative treatment was carried out. Following surgery, patients received oral analgesics after receiving an injection for two to three days. When the amount of fluid produced was below 300 mL, generally on the third postoperative day (POD), the nasogastric tube was withdrawn. Until oral fluids began, additional intravenous, or IV, fluids were administered. Most patients began taking oral fluids on the fourth or fifth postoperative day and they resumed their regular diets five to seven days after surgery. As per standard procedure, intravenous antibiotics were administered for five days following surgery. The length of the ileus, tolerance to a normal diet, vomiting, abdominal distension and signs of anastomotic leak were all observed in the patients. According to past records, patients experiencing stoma closure at our institute stayed an average of 10 days. In both instances, patients received treatment one day before the planned surgery. In order to evaluate the effect of early vs. typical closure on LoH, the evaluation of LoH was divided into three groups: those who remained for duration lesser than ten days, those who remained between ten and twenty days and those who remained for longer than twenty days. This study examined both surgical complications as well as medical complications the average number of complications in each category was used to compute the overall complication percentage. Following stoma closure surgery, patients were contacted for follow-up appointments at four weeks and three months.

## Statistical analysis:

The experimental data was placed in MS Excel and put for statistical analysis using SPSS version 21 software. Data was computed in the form of percentages. Chi square test was used for statistical analysis.

## Results:

Surgical complications like wound infection was lesser in ESC category (20%) when analysed against CSC category (34.54%). It was also observed that intra-abdominal collection was lesser in ESC category (14.0%) as compared to CSC category (20.0%). Anastomotic leak was also lesser in ESC category (4.0%) as compared to CSC category (9.1%). The findings were significant statistically (Table 1 - see PDF). The frequencies of different medical complications in both techniques of stoma closure are described in Table 2 (see PDF). Overall complications were lesser in ESC category (44.0%) as compared to CSC category (64.0%). Stoma related complications was also lesser in ESC category (4%) as compared to CSC category (6.0%) (Table 2 - see PDF). The proportion of patients with longer length of hospitalisation (10-20 days) was greater in CSC category (36.0%) as compared to ESC category (28.0%). Patients with < 10 days hospitalisation was greater in ESC category (70.0%) as compared to CSC (60.0%) (Table 3 - see PDF).

## Discussion:

Studies have shown that those with stomas had a worse quality of life than those who underwent similar treatments without developing a stoma. Reversing the temporary stoma enhanced quality of life [21, 22, 23-24], although acknowledging that the situation was temporary may make it more difficult to acclimate to managing a temporary ileostomy [13, 14, 15-16]. After intestinal resection, the primary function of a defuncting stoma is to protect the anastomosis and prevent pelvic infection [12, 13, 14- 15]. A Cochrane review found that less anastomotic leaks occur after provisional ileostomy. This study was conducted to compare early and conventional stoma closure following bowel surgery. In our study surgical complications like surgical complications like wound infection was lesser in ESC category (20%) when analysed against CSC category (34.54%). It was also observed that intra-abdominal collection was lesser in ESC category (14.0%) as compared to CSC category (20.0%). Anastomotic leak was also lesser in ESC category (4.0%) as compared to CSC category (9.1%). The findings were significant statistically. The findings of our study are having similarity with the findings of other studies [23, 24, 25, 26-27]. These studies also found lesser surgical complications in early surgical closure [24, 25, 26-27]. There are contradictory reports regarding early versus standard stoma closure [18, 19- 20]. However, there are substantial difficulties ranging from 0% to 9% and minor side effects ranging from 4% to 30% that can lead to a second procedure after stoma inversion [17, 18-19]. In our study, overall complications were lesser in ESC category (44.0%) as compared to CSC category (64.0%). Stoma related a complication was also lesser in ESC category (4%) as compared to CSC category (6.0%).The QoL was reported to better in patients with ESC. The observations of our study are supported by findings of other studies [23, 24, 25, 26-27]. Those studies also reported reduced frequency of medical complications in early closure [22, 23, 24-25]. A non-functional stoma reduces the need for an urgent reoperation, according a study [14, 15-16]. Repairing a stoma usually takes eight to twelve weeks. Nonetheless, stoma-related problems affect quality of life (QoL) during this period [17, 18-19]. Patients' discomfort and stoma-related problems may be reduced with early temporary stoma closure. Although there will be additional wound issues a study stated that a temporary stoma can be reversed 8-10 days after surgery [13, 14- 15]. Other studies found no appreciable variation in mortality and morbidity between stoma healing stages. A lower death rate is usually associated with intestinal continuity repair [21, 22- 23]. The proportion of patients with longer length of hospitalisation (10-20 days) was greater in CSC category (36.0%) as compared to ESC category (28.0%). Patients with < 10 days hospitalisation were greater in ESC category (70.0%) as compared to CSC (60.0%). The findings of our study are having similarity with the findings of other study [21, 22 and 27]. These studies also reported reduced stay at hospital in patients who underwent ESC [26, 27]. Ileostomies are still commonly carried out in emergency surgical settings in impoverished countries, where infectious diseases like gastrointestinal or tubercular punctures are common and patients present to doctors late in their illness, impeding primary closure [15, 16- 17]. Changes in lifestyle, sexual orientation and daily activities, as well as perceptions of a changed self-image, are among the many ways that patients respond to the formation of stomas [21, 22, 23-24]. Contrarily, stoma development is a treatment that improves health, alleviates pain and cures illness; it may even be advantageous [25, 26]. Early stoma closure does not increase the risk of surgery or hospitalisation, but it significantly improves the quality of life [27].

## Conclusion:

Early stoma closure is safe and it is a feasible procedure and does not raise the risk of surgical or medical problems.
